# Role of ultrasound in guiding the biopsy site in eosinophilic fasciitis

**DOI:** 10.2478/rir-2023-0032

**Published:** 2023-12-19

**Authors:** Felice Galluccio, Angelo Cassisa, Marco Matucci-Cerinic

**Affiliations:** Fisiotech Lab Studio, Rheumatology and Pain Management, Firenze, Italy; Morphological Madrid Research Center (MoMaRC), Madrid, Spain; Department of Anatomopathology, Ospedale San Giovanni di Dio, Florence, Italy; Unit of Immunology, Rheumatology, Allergy and Rare diseases, IRCCS San Raffaele Hospital, Milan, Italy

A 60 sixty-year-old woman presented with stiffness and pain in upper and lower limbs started 7 months earlier, followed by non-pitting edema in the forearms and legs extending centripetally. She also reports progressive asthenia with difficulty carrying out normal daily and work activities. Nothing significant in the medical history, no known family history of rheumatic diseases. Acute-phase reactants, autoantibodies, and leukocyte formula were normal and only an increase of β2-microglobulin (5.29 mg/L normal range 0.97–2.64) and gammaglobulin (24.4% normal range 10.6–18.8) with biclonal IgG chains was detected.

**Figure 1:**
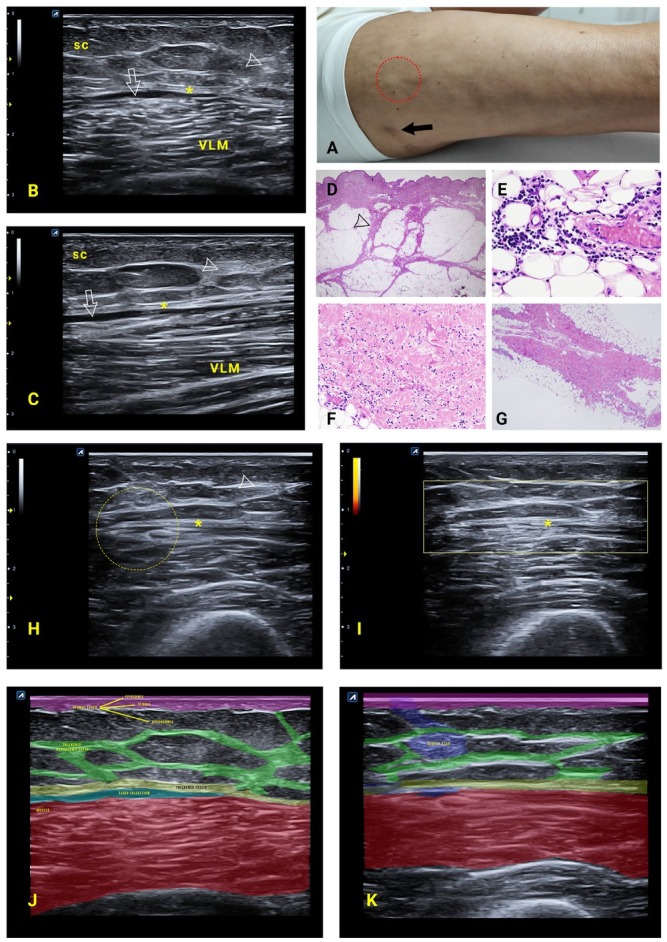
A. Patient’s right thigh; Black arrow: previous biopsy site; Red dotted circle: biopsied area corresponding to ultrasound findings. B-C. Ultrasound transverse (B) and longitudinal (C) scans; sc: subcutaneous tissue; *: thickening of the fascia; VLM: vastus lateralis muscle; arrowhead: thickening of hypodermic septa; white arrow: fluid collection between fascia and muscle. D-E-F-G. Histology findings: thickened hypodermic septa (arrowhead), lymphoplasmacytic, and eosinophils (E-F-G) on thickened fascia (F-G). H-I. Post-treatment ultrasound transverse scan; dotted circle: biopsied area. *: fascia and resolution of perifascial fluid collection; arrowhead: note the reduction of the thickness of the hypodermic septa and of the subcutaneous tissue; negative PD signal. Colorized versions of ultrasound images (J) before and (K) after treatment, demonstrating the echostructure improvement useful for patient monitoring.

The thigh biopsy (Figure 1A, black arrow) detecting only a minimal inflammatory infiltrate and sparse collagen fibers, did not allow any specific diagnosis. The ultrasound (US) scanning of the vastus lateralis muscle (Figure 1B and 1C, vastus lateralis muscle (VLM)) identified a diffuse thickening of the fascia (Figure 1B and 1C, asterisk) and hypodermic septa (Figure 1B and 1C, arrowhead), as well as focal areas of fluid separation of the fascia from the surrounding tissues (Figure 1B and 1C, white arrow): the area was hyperechoic with a loss of muscle echostructure, due to focal areas of greater inflammatory involvement. The new biopsy, performed in this area (Figure 1A, red dotted circle), showed a fascial thickening (Figure 1F and 1G) with hypodermic septa (Figure 1D, arrowhead), and a lymphoplasmacytic infiltrate characterized by an eosinophilic accumulation (Figure 1E, 1F and 1G), thus suggesting the diagnosis of eosinophilic fasciitis.^[1]^

The patient was treated with methylprednisolone sodium succinate 500 mg intravenously (IV) pulses for three consecutive days, followed by oral methylprednisolone 16 mg/day combined with methotrexate 10 mg/week. Pulsed therapy with steroids was chosen to try to block the extension of the disease more rapidly, which had also affected the trunk and neck. After two months of treatment, despite the progressive reduction of cortisone up to 4 mg/day, the altered values of gamma globulins (17%) and 2-microglobulin (2.40 mg/L) normalized. The area of interest was followed up with US, showing the resolution of the perifascial fluid collection [Figure 1H-1I asterisk], the reduction of the thickness of the hypodermic septa [Figure 1J and 1K] and of the subcutaneous tissue. This case highlights the role that US may have in identifying the site where to perform the biopsy in a patchy disease distribution like fasciitis. Thus, US may help either in the diagnostic procedure to choose an area of disease activity and in the follow up to evaluate directly at patient bedside the therapeutic efficacy.^[2]^ Among imaging methods, magnetic resonance imaging (MRI) is probably superior in detecting abnormalities of eosinophilic fasciitis,^[3]^ but ultrasound is superior overall because it is cost-effective, has virtually no contraindications and is certainly much better accepted by patients, being able to use it from diagnosis to biopsy guide up to the entire follow-up.
